# Synthesis and thermochromic property studies on W doped VO_2_ films fabricated by sol-gel method

**DOI:** 10.1038/s41598-017-05229-9

**Published:** 2017-07-21

**Authors:** Guoping Pan, Jinhua Yin, Keli Ji, Xiang Li, Xingwang Cheng, Haibo Jin, Jiping Liu

**Affiliations:** 1 Beijing Key Laboratory of Construction Tailorable Advanced Functional Materials and Green Applications, School of Materials Science and Engineering, Beijing Institute of Technology, Beijing, 100081 China; 2 Physics Department, University of Science and Technology Beijing, Beijing, 100083 China

## Abstract

Tungsten-doped VO_2_ thin films have been synthesized by a modified sol–gel process and followed by a post annealing. Vanadium pentoxide and tungstic acid as raw materials with the addition of hydrogen peroxide, concentrated hydrochloric acid (catalyst) and oxalic acid used as reducing agent were reacted in isobutanol. Finally, the uniform sol of vanadyl oxalate in isobutanol solvent was obtained as precursor. Detailed study suggested that W doped in VO_2_ introduces additional electron carriers and induces the formation of V^3+^. Post annealing under vacuum promotes the releasing of chemical stress and generates oxygen vacancies in the samples. Temperature dependent transmittance study revealed that the releasing of chemical stress and deliberately introducing oxygen vacancies in W-doped VO_2_ films have positive effects on enhancing its switching ability in the infrared transmittance as the metal-insulator transition (MIT) occurs. The largest switching of transmittance was obtained about 48% in the infrared range at 43 °C in 1.5%W doped VO_2_ films, which is significantly larger than the reported ones. The findings in this work open a new way to synthesize the novel and thermochromic W doped VO_2_ films with facility and low cost. Therefore, it has extensive application to construct smart windows and electronic devices.

## Introduction

Vanadium dioxide has attracted widespread interest since the discovering of its metal-insulator transition (MIT) in 1959s^1^. It is well-known that the MIT in VO_2_ is a first-order reversible transformation from its insulating monoclinic phase (P2_1_/c) with an energy gap of 0.7 eV at low temperature to metallic tetragonal phase (P4_2_/mnm) at high temperature with the critical temperature (T_c_) of 68 °C^1–3^. The theoretical explanation of the MIT was presented by Goodenough^4^ that the t_2g_ band of VO_2_ is splitting into d_II_ band and the π^*^ anti-bond in the framework of crystal field theory which was verified by structural simulation calculations and experimental results^5–7^. There are significant changes in the electrical resistivity and infrared transmittance along with the occurring the MIT. Infrared light would go through VO_2_ films mostly whereas it would be reflected above 68 °C with the same transmittance of visible light. So VO_2_ has potential applications in sensors, optical storage device, infrared modulators and intelligent window coating^8, 9^.

In view of applications, the T_c_ of VO_2_ should be reduced, while the switching ability of its transmittance in infrared should be kept or enhanced during the MIT. In spite of extensive efforts contributed, these problems are still unsolved, which are crucial for the application of VO_2_^8, 10, 11^. Transition metal elements with large atomic radius and rich valences introduced into VO_2_ could trigger the distortion of its lattice and modify its properties related to MIT. Reducing T_c_ of the MIT could be achieved efficiently by the incorporation of some metal ions, such as W^6+^, Mo^6+^, and Nb^5+^, into the crystal lattices of VO_2_^3, 10–12^. W has been proved to be most effective dopant in reducing the T_c_ by 21–28 °C/at.%^13, 14^, which has been confirmed in the study on VO_2_ films prepared by various methods including CVD, sputtering and sol-gel^13–16^. However, W doping in VO_2_ results in the increase of its electron intensity, which would lead to weakening of its revisable optical switching properties during the MIT^13, 14^. On the other hand, holes introduced into VO_2_ could improve its revisable switching properties both in resistivity and transmittance in infrared efficiently^17^. Therefore, elaborately introducing holes into W-doped VO_2_ could realize the reducing of its T_c_ and improve its revisable switching properties during its MIT effectively. Holes can be introduced by deliberately controlling the oxygen vacancies in VO_2_ films, which can be achieved by post annealing samples in high vacuum. Oxygen vacancies in W-doped VO_2_ films could be an unexpected route to approach VO_2_ the sample with low MIT temperature and strong ability to switch both conductivity and infrared transmittance^18^.

As for the preparation of VO_2_ films, sol-gel method has been demonstrated to be an effective skill and it is relatively cheap and can be scaled to large areas. In the case of the fabrication of VO_2_ films by sol-gel, oxalic acid and alcohols are commonly used as reducing agents to prepare the precursor in the aqueous or methanol solvent^19, 20^. The interaction between sol and precursor has a significant effect on the morphology of the pre-deposited film. Therefore, it is required that the surface of the substrate, for example, silicon substrate, should be subjected to hydrophilic treatment essentially (Details can be seen in the Supplementary Information). For the alcohol reducing agent such as isobutanol^21^, vanadium pentoxide could be reduced to vanadium isobutoxide (VO(i-OC_4_H_9_)_2_, which needs to be carried out at more than 100 °C due to their weak reducing property. These factors would lead to the complexity of the thin film preparation process. Therefore, new skills and approaches for the synthesis of precursor sols and fabrication of pre-deposition thin films are needed.

In this work, a modified sol-gel method was developed to fabricate VO_2_ thin films. In brief, a certain ratio of V_2_O_5_ and oxalic acid were added to amounts of isobutanol solvent, and uniform sol of vanadyl oxalate was obtained at 70 °C. To introduce W ions into VO_2_ in this case, tungstic acid was selected and solved in excessed hydrogen peroxide to form a complex solution firstly, and then injected into isobutanol solution that contains oxalic acid and vanadium pentoxide to prepare W-doped precursor. The remaining hydrogen peroxide in the W-doped precursor could promote the reduction of vanadium pentoxide owing that O_2_^2−^ bonds as ligand coordinated with V^5+^ to obtain (VO(O_2_)_2_)^−^^22^. Finally, transparent precipitation-free sols with W doping would be obtained and the whole process is more efficient and economic as compared with the direct use of vanadium acetylacetonate and tungsten chloride^23^.

After then, pre-deposited thin films with high uniformity were formed by spin-coating and drying. Compared to aqueous, methanol and isopropanol sols, isobutanol sols of vanadium oxalate can be spin-coated on the surface of sapphire substrate to form uniform and smooth thin films without hydrophilic treatment. Followed by annealing under high vacuum conditions (~10^−3^ Pa), the undoped and W-doped VO_2_ films were fabricated. For the undoped films, the phase transition temperature is significantly reduced below 68 °C with decent infrared performance. According to previous reports, W doping in VO_2_ induces its MIT temperature decreasing, however, its infrared characteristics faded^12, 13, 23^. In this paper, the infrared performance of W-doped VO_2_ films annealed under vacuum conditions have been significantly improved while the phase transition temperature is reduced, which has not been reported in the previous work. These properties make W-doped VO_2_ films studied in this work meet the requirements in realizing the practical application.

## Experimental

All the chemicals used in this work, i.e., V_2_O_5_ (99.9%), H_2_WO_4_ (99.3%), H_2_O_2_ (30 wt%), HCl (37.5 wt%) and H_2_C_2_O_4_ · 2H_2_O (99%), were utilized without any further purification. The c-cut (0001) Al_2_O_3_ was used as substrate to grow VO_2_ films.

## Preparation of precursor solution

First, 28 mg, 42 mg, and 56 mg H_2_WO_4_ were dissolved in 3 mL 30 wt% H_2_O_2_ by ultrasound respectively and the three transparent solutions were obtained. Next, these H_2_WO_4_ solutions were poured into three 100 mL beakers including 40 mL isobutanol solvent under stirring for several minutes at room temperature. Then, 1 g V_2_O_5_, 3 mL 36 wt% HCl and 1.26 g H_2_C_2_O_4_ · 2H_2_O (with the mole ratio of V_2_O_5_: H_2_C_2_O_4_ · 2H_2_O = 1:2) were added into the three systems, and then followed a stirring thermostatically in the water bath at 70 °C for around 9 h. Finally, the three blue-green isobutanol sols of VOC_2_O_4_ with 1 at.%, 1.5 at.%, and 2 at.% W-doping were obtained.

## Preparation of VO_2_ film

The c-cut Al_2_O_3_ substrates were cleaned ultrasonically consequently in ethanol, acetone and No.II cleaning fluid (NH_3_ · H_2_O:H_2_O_2_:H_2_O = 2:2:5, molar ratio)^24^ to remove organic contaminations on the surface. The deposition was carried out by spin coating method, and precursor films were formed on the cleared c-sapphire substrates with a spin speed of 600 rpm/30 s and 4000 rpm/40 s. Then, the films were dried at 60 °C for 20 min. This process was repeated several times to obtain expected thickness. Finally, the pre-deposited films were annealed at 450 °C, 470 °C and 500 °C for 4 hours respectively in a furnace under the vacuum of 10^−3^ Pa, with the heating rate of 5 °C/min. As a comparison, VO_2_ films were also fabricated by aqueous and alcohol Sol-Gel, and the morphology of the corresponding samples was recorded by SEM. The detailed results are shown in Figure S1 in the Supplementary Information. Figure S1a–c are the morphology of VO_2_ films synthesized by aqueous Sol, alcohol Sol and isobutanol Sol, respectively. It is clear to see from Figure S1a that the morphology of VO_2_ produced from aqueous Sol is discontinued or isolated islands, the morphology of VO_2_ film produced from alcohol Sol is quite rough with some isolated larger grains distributed on the surface, and the morphology of VO_2_ film produced from isobutanol Sol is much uniform and smooth. These results demonstrated that employing isobutanol Sol to produce VO_2_ films has prominent advantages.

## Characterizations

The crystalline structure of the films was determined by X-ray diffraction (XRD, Bruker-AXS diffractometer, Model D8 ANVANCE) with Cu-Kα radiation source. 2θ mode was used to scan all the samples in the range of 15°~50° with the step of 0.02° and the stay time is 2~3 s. Morphology was characterized by atomic force microscope (AFM, Bruker-Nano scope Multimode IIIa) by tapping mode. Raman spectrum was measured to determine the lattice vibration of the films by Microscopic confocal Raman Spectrometer (HR800, excitation wavelength: 633 nm, laser power: 1 mW) and the integration time is 80 s. The chemical valences of constituents were measured by X-ray Photoelectron Spectroscopy (XPS) (PHI QUANTERA-II SXM) with Al-Kα radiation source (1486.6 eV). The optical properties of the films were investigated by spectrophotometer (Nicolet iS50 Fourier Transform Infrared Spectrometer) to analyze the transmittance of the films in the wavenumber range of 400–4000 cm^−1^ on heating and cooling. The device provides a solid heating accessory that can test the infrared transmittance of the sample under variable temperature conditions. The light source is ETC Everglo. Hysteresis loops were obtained by collecting the transmittance of films at a fixed wavelength (2500 nm) at approximately 2 °C intervals in the temperature range of 24–90 °C.

## Results and Discussions

**Figure 1:**
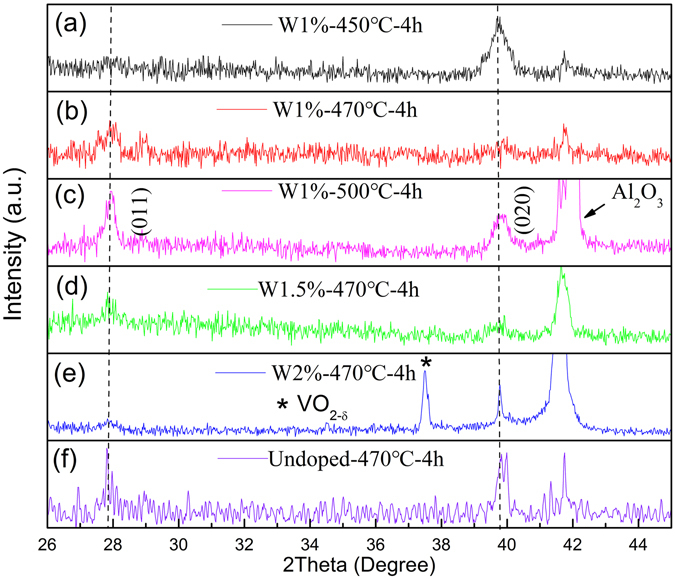
XRD patterns of VO_2_ films grown on sapphire substrates, (**a**–**c**) annealed at 450, 470 and 500 °C for 4 h with nominal 1 at.%W-doping; (**d**–**f**) annealed at 470 °C for 4 h with nominal 1.5 at.%, 2 at.%W-doping and undoping. The dashed lines are guide for the eyes.

Figure 1a~c show the XRD patterns of the VO_2_ films with 1%W doping and sintered at 450, 470, and 500 °C, respectively. As can be seen from Fig. 1a, in addition to the peak of Al_2_O_3_ (0006), only one peak at 39.9° was observed and can be indexed to (020) crystallographic plane of VO_2_, similar with previous reports^18, 24^.

For the samples annealed at 470 and 500 °C, however, two dominant diffraction peaks at 27.8° and 39.9° appeared, which can be indexed to VO_2_ (011) and (020) planes. The intensity of (011) diffraction peaks gradually increased as the annealing temperature increased, which is in well agreement with the reported results^25^. Therefore, Fig. 1a–c reveal that the orientation of the 1% W-doped VO_2_ grown on Al_2_O_3_ (0006) is strongly dependent on the annealing temperature. The relative low-annealing temperature favors the growth of (020) orientation for VO_2_ film, while relative high-annealing temperature induces the dominant orientation of (011) of corresponding samples.

Figure 1d~f show the XRD patterns of the VO_2_ films with undoped, nominal 1.5% and 2% W-doped samples annealed at 470 °C/4 h, two prominent peaks at 27.8° and 39.8° were observed and can be indexed to (011) and (020) planes. However, one additional diffraction pattern at 37.4° appeared in the 2% W-doped samples, which can be assigned to VO_2-δ_, suggesting second phase was formed in this sample, agreed with previous report^18^. Therefore, Fig. 1 indicated that the samples were grown with (011) and (020) orientation on the c-Al_2_O_3_ substrate, no epitaxial growth behavior observed^26^.

**Figure 2:**
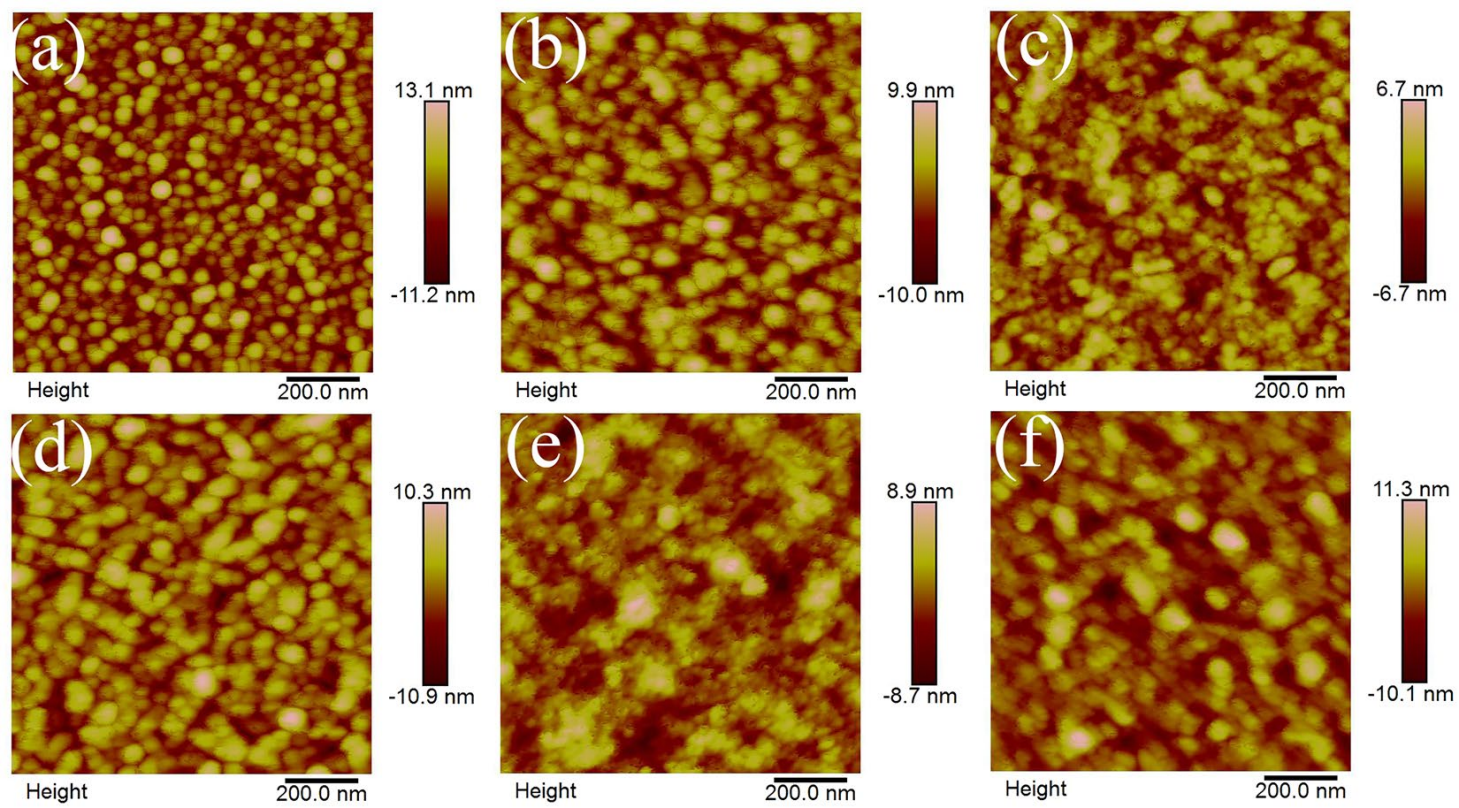
AFM images of VO_2_ films of nominal 1%, 1.5% and 2% W doped samples. (**a**~**c**) annealed at 470 °C/4 h; (**d**~**f**) annealed at 500 °C/4 h.

Figure 2 shows AFM images of VO_2_ films with nominal 1%, 1.5% and 2% W doping. a)~c) annealed at 470 °C/4 h; d)~f) annealed at 500 °C/4 h. The morphologies of the films are granular structures with grain size in range of 60–80 and 70–110 nm for 470 and 500 °C annealed samples, respectively.

Compared samples annealed at 470 °C, it is clear to see that with increase in W contents, the surfaces of the corresponding samples become more impacted with average grain size reduced gradually, but the grain size distribution broadened. Meanwhile, for the samples annealed at 500 °C, the grain size of 1% W doped sample is significantly larger than that annealed at 470 °C. Moreover, with increase in W doping content, the grain sizes of the samples reduced with a similar tendency of these annealed at 470 °C. However, the agglomeration of grains is distinct, which induces the grain size distribution widened drastically. Another characteristic of the surface morphologies result is that increasing the doped W content or increasing annealing temperature induces the surface of the samples became more softened, which suggested that some downy or porous structures were formed on the surface of the grains, consistent with previous study by SEM^27^.

To determine the thickness of samples, the cross section image was performed by SEM and it was found that all the samples are about 300 nm. As evidence, the cross section image of 1% W-doped VO_2_ films annealed at 470 °C/4 h is presented in the Figure S2 in the Supplementary Information.

**Figure 3:**
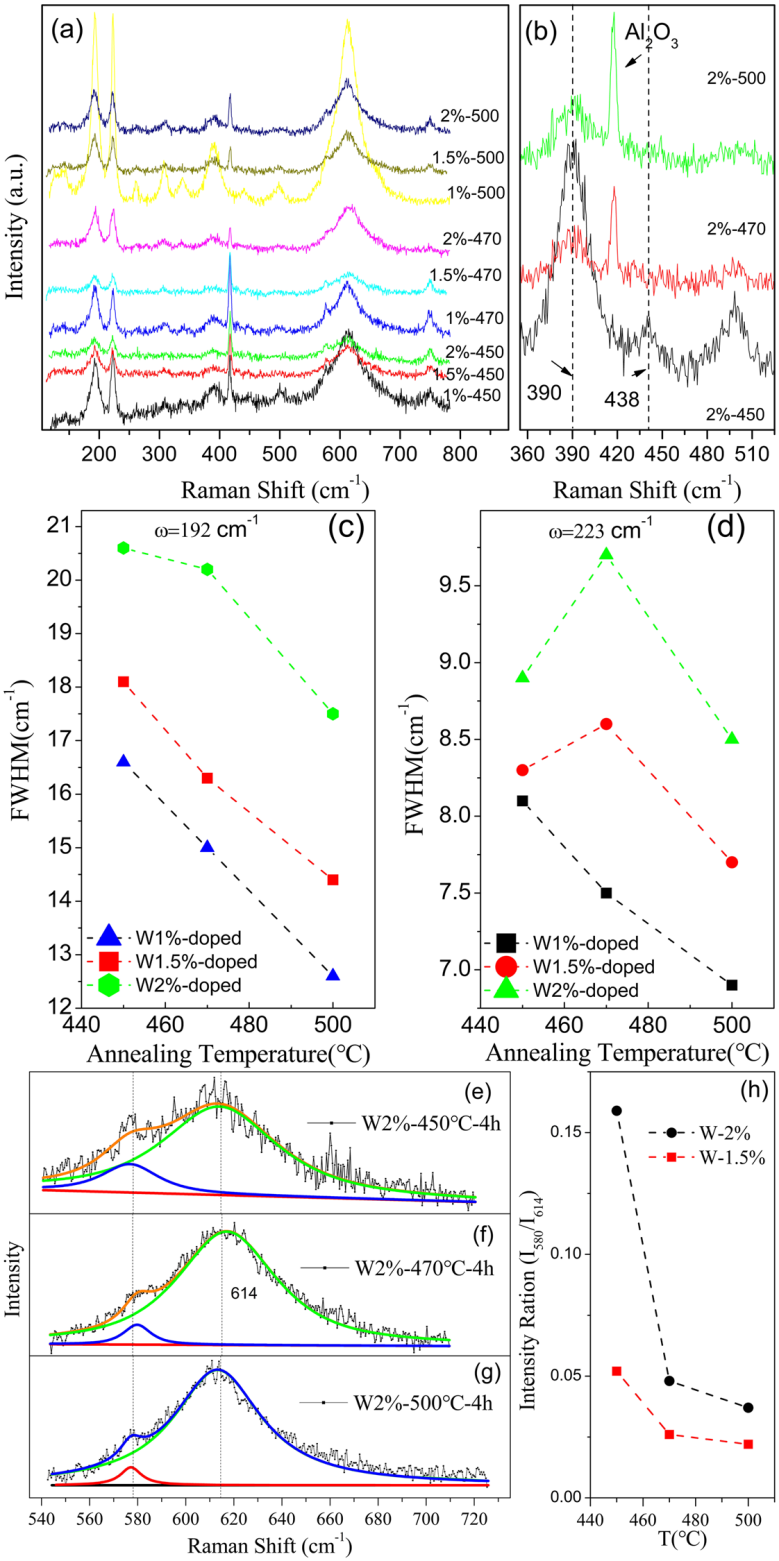
(**a**) Raman spectra of the films annealed at 450, 470 and 500 °C; (**b**) The magnified Raman spectra in the range of 350~520 cm^−1^ for 2% W-doped samples annealed at different temperatures; (**c**,**d**) the annealing temperature dependent FWHMs of the peaks at 192 and 223 cm^−1^; (**e**~**g**) the V-O related vibration mode positions of films with nominal 2 at.% W annealed at 450, 470 and 500 °C; (**h**) the annealing temperature dependent integrated intensity ratio of peaks at 580 and 614 cm^−1^.

Raman microscopy is considered to be an effective skill to calibrate the bond vibrations in the lattice of VO_2_^11, 27, 28^. The peaks at 192 (ω_V1_) and 223 (ω_V2_) cm^−1^ in Raman spectrum of M1 phase VO_2_ are connected with the V-V bond vibrations, while the peak at 614 cm^−1^ is involved with the vibration of V-O bonds in VO_2_^11, 29^. The characteristics of these Raman peaks are very sensitive to the chemical states of the ions bonded in VO_2_ lattices^30–32^. Consequently, the substitution of V to W in VO_2_ lattice should affect the V-V(W) and V(W)-O bonds states, thus, the corresponding Raman vibration states should be modified, as well^11, 28, 32^. Figure 3a shows room temperature Raman spectra for nominal 1%, 1.5% and 2% W-doped samples annealed at 450, 470 and 500 °C, respectively. In addition to the weak peaks of substrate at 417 and 751 cm^−1^, the peaks at 192, 223, 264, 307, 338, 387, 440, 498 and 611 cm^−1^ in Fig. 3a can be assigned to the monoclinic VO_2_^8, 28–31^. Fig. 3b is the magnified Raman spectra in the range of 350~520 cm^−1^ for 2%W-doped samples annealed at different temperatures. Compared with the Raman spectra of different samples, an interesting result is that peak located at 438 cm^−1^ was observed in the sample annealed at 450 °C, which can be assigned to T-phase VO_2_ and can be a critical evidence for the existing of stress in the studied sample^28, 31^. However, this peak disappeared in samples annealed at 470 and 500 °C, which suggests that T-phase VO_2_ or stress was reduced in the corresponding samples.

Figure 3c,d are annealing temperature dependent full width at half maximum (FWHM) of peaks at 192 and 223 cm^−1^ for samples with different W doping. Figure 3c,d reveal that higher W content sample has larger FWHM of its Raman peaks; meanwhile, with increase of annealing temperature, the FWHMs of 192 and 223 cm^−1^ peak gradually decreased. These results illustrate that W introduced into VO_2_ induces its V-V involved Raman peaks broaden, which further suggests W doped in VO_2_ gave rise to additional stress randomly in the corresponding samples^11, 28^. However, samples annealed at higher temperature have narrowed FWHMs of the Raman peaks at 192 and 223 cm^−1^, which suggests that distortion or stress in the VO_2_ lattice degenerated, consistent with previous reports^11, 28, 31^.

Figure 3e,f are the magnified Raman spectra in range of 530~730 cm^−1^ with fitting results also shown in detail. Figure 3e,f suggest that one main peak located at 614 cm^−1^ and a weak shoulder located at 580 cm^−1^, the former one is belong to M1 phase VO_2_, and the later one is considered as an evidence for the existing of T-phase VO_2_ or the appearance of stress^29–32^. The fitted integrated intensity of these peaks for samples with nominal 1.5 and 2% W doping contents and annealed at different temperature are graphed in Fig. 3h.

Figure 3h indicates that increasing in doped W content, the stress of the produced samples enhanced remarkably. While the integrated intensity ratio of peaks at 580 and 614 cm^−1^ are drastically reduced with annealing temperature increasing, which suggests that the stress is reduced intensely in the corresponding samples^32^.

Therefore, the results in Fig. 3 demonstrate that increasing in annealing temperature leads to the growth of crystal grains, promotes W diffusion and eliminates its disordered distribution in VO_2_ lattice. Finally, the crystal quality of the corresponding samples improved with reduced doping related stress, consistent with our XRD study.

**Figure 4:**
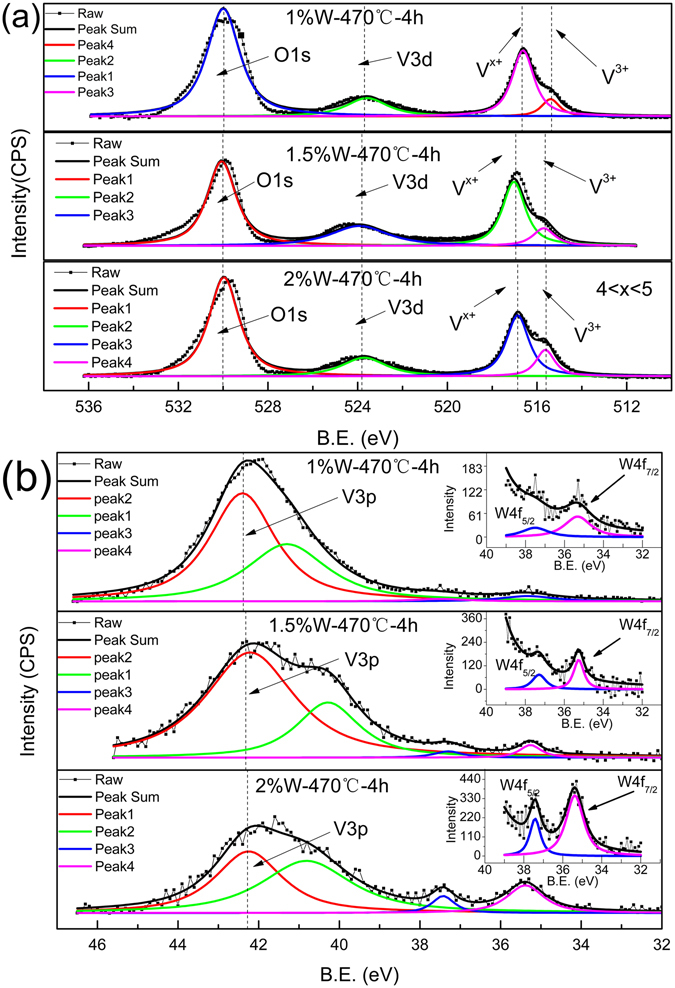
XPS survey spectrum of nominal 1 at.%, 1.5 at.% and nominal 2 at.%W -doped VO_2_ annealed at 470 °C: (**a**) V2p3; (**b**) W4f. The dashed lines are guide for the eyes.

The XPS was performed to determine the constituents and element chemical states. Figure 4 is the XPS spectra of films with 1%, 1.5% and 2%W doping and annealed at 470 °C. Figure 4a suggests that O1s peak is located at 530.3 eV, and two distinct peaks of V2p3 at 517.3 eV and 515.9 eV. In the light of the results, the ΔE difference between O1s and V2p3 is 13 eV a little lower than 13.4 eV on previous studies^33–35^, which could be attributed to V^x+^(x defines as the mixture of valence between V^4+^ and V^5+^ with dominating V^4+^) and V^3+^, respectively. Considering the characterization of XPS skill, the presence of fully oxidized vanadium could be originated from the partially oxidized surface^36, 37^ and also confirmed by surface etching^23^.

Figure 4b shows the XPS spectra of V and W ions, the insert to Fig. 4b is the details of the XPS spectra of W(4f_5/2_) and W(4f_7/2_). It is clear to see that binding energies of W(4f_5/2_) and W(4f_7/2_) are located 37.3 eV and 35.3 eV, conforming that the chemical valences of W ions are + 6. These results are consistent with the previous reports^38^. Compared with XPS spectra of different films, one can see that with increase of W content, the integrated intensity of V^3+^ gradually increased compared with that of V^x+^. This result implies that W doping in VO_2_ denoted additional electrons that could have reduction effects on the neighbored V ions, which is the intrinsic reason for the increasing of the density of V^3+^, and finally, will induce the formation of VO_2-δ_, as demonstrated by our XRD results and previous studies^39, 40^.

Table 1 lists the integrated intensity ratios for V2p/W4f and V^x+^/V^3+^, which are extracted from XPS result in Fig. 4. Table 1 indicates that as the nominal doped W content increased, the integrated intensity ratio of W to V enhanced, suggesting W ions doped in the lattice VO_2_.

**Table 1:** Intensity ratios of (V2p)/(W4f) and (V^x+^)/(V^3+^) for films annealed at 470 °C according to the XPS survey spectrum.

Samples	(V2p)/(W4f)	(V^3+^)/(V^x+^)
Nominal 1 at.%W	1: 0.007	1:4.73
Nominal 1.5 at.%W	1: 0.01	1:3.84
Nominal 2 at.%W	1: 0.036	1:2.83

However, the intensity of VO_2-δ_ related peak observation in Fig. 1b disagrees with the measured XPS results in Fig. 4, which means that large domains with much reduced V formed in samples annealed at 500 °C. Previous studies revealed that inject high energy electrons into VO_2_ results in the reducing of V^4+^ to lower chemical states and generates oxygen vacancies^25^. Specially, the appearance of oxygen vacancies in VO_2_ could give raise to the relaxation of the chemical stress^32^. The results of Raman and XPS spectra study in this work support this idea indeed. Therefore, it is reasonable to postulate that there are plentiful oxygen vacancies in the studied samples, which results in some V^4+^ ions were reduced to V^3+^, consistent with previous temperature dependent resistance studies^41, 42^. Another reason for the generation of oxygen vacancies could be the escaping of oxygen from samples owing to annealing at high temperature under high vacuum. This postulation agrees with the Raman study results shown in Fig. 3, and is similar with the report by R. A. Aliev *et al*., where the authors demonstrated that the vanadium dioxide was reduced to V^3+^ as the oxygens escaped into a vacuum and a nonstoichiometric oxygen-poor vanadium dioxide films formed^18^.

**Figure 5:**
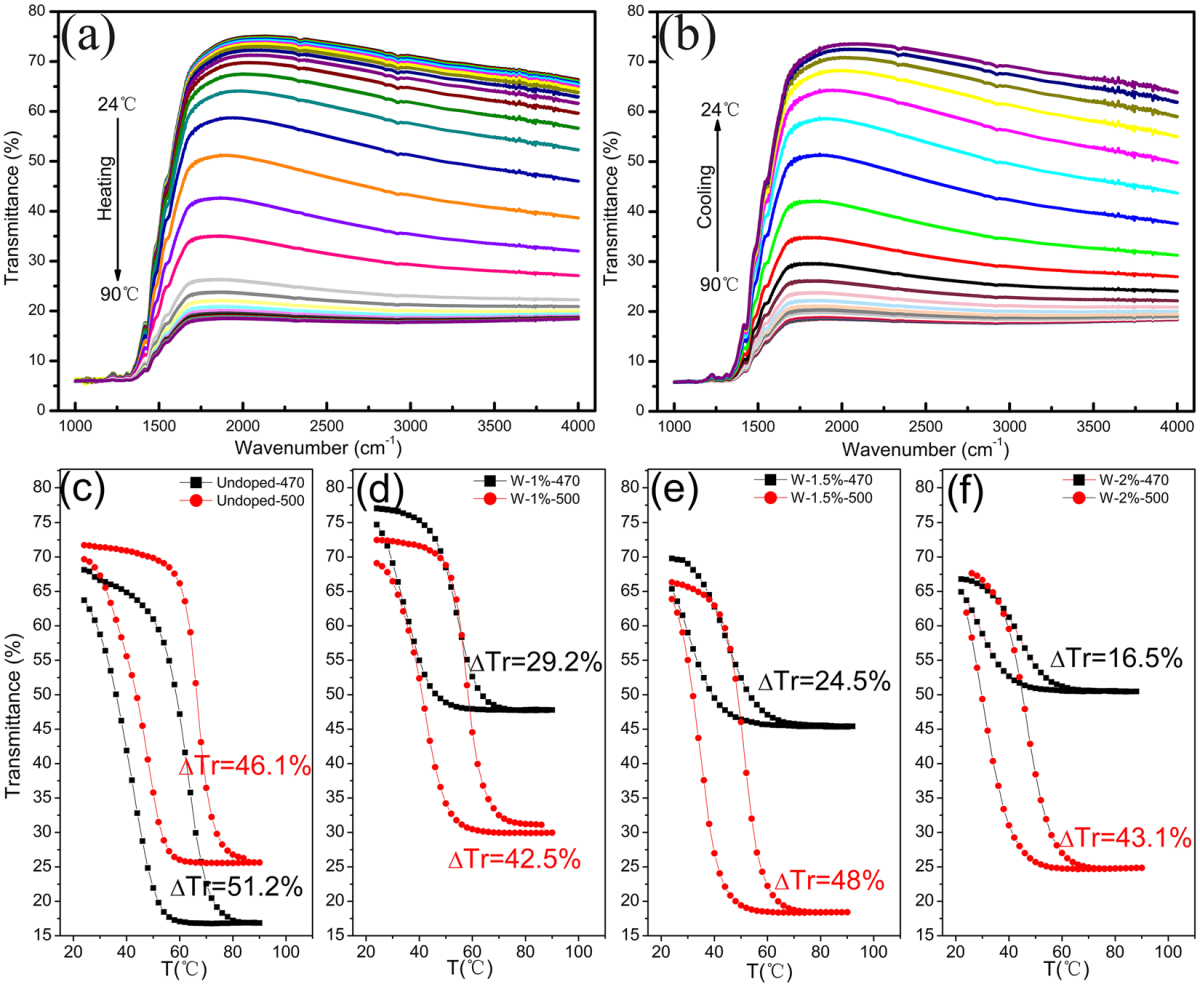
The infrared transmittance curves of the 1.5%W doped samples in the (**a**) heating and (**b**) cooling in the range of 1000–4000 cm^−1^. Hysteresis loops for the temperature dependent transmittance of (**d**) Undoped; (**d**) nominal 1 at.% W doped; (**e**) nominal 1.5 at.% W doped; (**f**) nominal 2 at.% W-doped VO_2_ films annealed at 470 and 500 °C.

**Figure 6:**
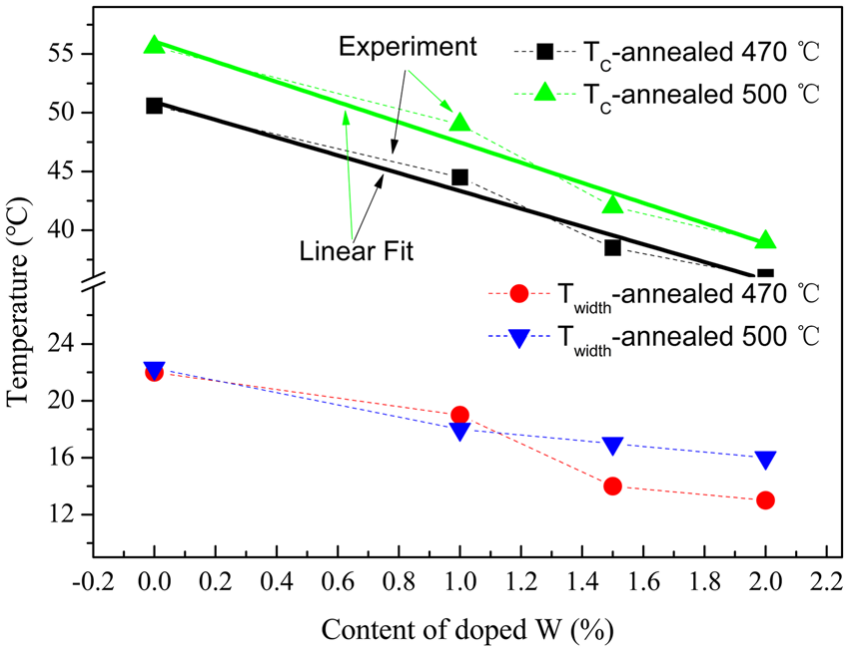
The MIT switching temperature (T_c_) and width of the transmittance switching relaxation (T_width_) of samples with different W doped content, and annealed at 470 and 500 °C.

Figure 5a,b are the temperature dependent transmittance (T_r_) curves of 1.5%W doped sample recorded in 1000~4000 cm^−1^. The transmittance curves were measured in range of 24~90 °C with interval 2 °C. A series of infrared transmittance curves were obtained throughout the process upon heating and cooling, respectively. Figure 5a indicates that with temperature increasing from 24 to 90 °C, the transmittance of the studied sample gradually reduces in the measured infrared range, vice versa as indicated in Fig. 5b. These results demonstrate that a reversible MIT occurred, consisting with previous studies^43^. The temperature dependent transmittance of undoped, 1%, and 2% W-doped VO_2_ films annealed at 500 °C/4 h samples upon heating and cooling branches can be seen in the Figure S3 in the Supplementary Information. From the Figure S3, we can see that all the samples have the similar trends with that of 1.5% W-doped VO_2_. Fig. 5c~f are hysteresis loops of temperature dependent transmittance at 4000 cm^−1^(~2500 nm) obtained by extracting the transmittance at a fixed wavelength (2500 nm) at approximately 2 °C intervals for samples with undoped, nominal 1 at.%, 1.5 at.% and 2 at.% W-doped VO_2_ films annealed at 470 and 500 °C, respectively. The obvious switching in IR transmittance was observed in all samples. W-doped films annealed at 470 °C give low IR transmission switching ability (ΔT_r_) of 29.2%, 24.5% and 16.4% (defined as ΔT_r_ = T_r24 °C_-T_r90 °C_) in spite of low transition temperatures (T_c_) that is determined by (T_c-heating_ + T_c-cooling_)/2, in which T_c-heating_ and T_c-cooling_ are the maximums of the dTr/dT in the heating and cooling cycle, consistent with Nb doped VO_2_ studies^44^. The ΔT_r_ was increased drastically after increasing the annealing temperature to 500 °C, which are 42.5%, 48%, and 43.1% for 1%, 1.5% and 3% W doped samples, with slightly increasing transition temperatures, respectively. The details of W content dependent T_c_ and T_width_ are graphed in Fig. 6, which indicates that with increase of W doping content, the T_c_ and T_width_ of the corresponding samples decreased; meanwhile, increasing annealing temperature induces the T_c_ and T_width_ of the corresponding samples increased. The T_c_ transforms 50.6 °C for undoped VO_2_ films to 36 °C for 2% W-doped films annealed at 470 °C/4 h (from 55.6 °C to 39.1 °C for films annealed at 500 °C/4 h) and decreases linearly with doping concentration. The solid lines are a linear fitting. The changed ratio of T_c_ is estimated to be about −7.5~−8.5 °C per 1 at.% of W doping and this change is lower than 21~28 °C/ W at.% but comparable to the value by another group^45, 46^. The lower rate of change in the critical temperature ΔTc as a function of W-doping concentration is a weakness in the studied samples in this work; however, Sol-Gel processing is simple, low-cost and productive. The much interesting result is that attenuated infrared characteristics induced by W-doping were enhanced remarkably, which were not achieved by conventional sputtering or pulsed laser deposition to date.

In this work, all samples were annealed under the vacuum of 10^−3^ Pa and the resulting oxygen vacancies could weaken the decreasing trend induced from W-doping. The vacancies may act as the heterogeneous nucleation sites for the phase transition of undoped and W-doped VO_2_ films. Furthermore the phase change activation energy of MIT for undoped VO_2_ films could be lowered so the MIT temperature of undoped VO_2_ films was decreased far below 68 °C^18, 42^.

According to our Raman spectra study, with increasing in the content of W, Raman vibration peaks of the produced samples broadening, which implies its chemical stress enhanced due to the differences of ion radius between W^6+^(0.062 nm) and V^4+^(0.058 nm). The enhanced stress has been clarified of negative effects on the IR switching ability, which also confirmed by the variation of transmittance during the MIT^11^. However, with increasing annealing temperature, the chemical stress in samples with different W contents is reduced in spite of its grain size enhanced. This result could be attributed to compensative effect of the additional electrons carriers and generated oxygen vacancies as proved by our Raman and XPS spectra studies, which is consistent with previous reports^18, 44, 47, 48^.

Previous studies suggested that introduction of W would break V^4+^-V^4+^ bonds and the new V^3+^-W^4+^ and V^3+^-V^4+^ bonds formed and result in nonstoichiometric vanadium oxides, which destabilizes the semiconducting phase of VO_2_ and lowers the transition temperature of VO_2_ films from monoclinic phase to tetragonal phase^14, 16^. Recently, studies evidenced that W replaces V in VO_2_ lattice leading to local stress in VO_2_, which results in the decrease of the activation energy for the M-R phase transition, thus, reduce the MIT temperature^39, 40^. Furthermore, the existing of the internal or external stress on M phase VO_2_ causes its switching ability of infrared wave band deteriorating^13, 23, 49^. Accordingly, W doping in VO_2_ forms some W-rich domains, which results in the MIT temperature of different domains is different, thus, the global MIT temperature of samples much widened. As the temperature of samples rising, these W doped domains will gradually transfer to R Phase VO_2_, and finally percolation structures formed, consequentially, the width of the MIT broadened. Likewise, rising annealing temperature promotes the diffusion of W in VO_2_, which will smear the rich W domains and unify the distribution of W in VO_2_. In this way, the energy barrier for MIT enhanced, which will lead to the increase of the phase transition temperature of MIT with narrowed temperature width of its relaxation. This postulation indeed agrees the characteristics of the temperature dependent transmittance loops as shown in Figs 5 and 6. The optimized MIT properties was realized at nominal 1.5% W doped VO_2_ films annealed at 500 °C with MIT temperature about 43 °C and the variation of infrared transmittance about 48% in this work, and this result is the reported highest switching ability in IR transmittance as the MIT occurred. Another interesting result in Fig. 5 is that as the doped W is about 2%, plentiful second phase, namely VO_2-δ_, was formed as corroborated by the XRD and XPS studies in this work. The formation of second phase drastically gives rise to the degeneration of its switching ability about the infrared transmittance in the produced samples.

Based up the above results and analysis, modification of the electron carrier density by doping W and introducing oxygen vacancies would effectively realize the controlling of the MIT temperature and the switching ability of IR transmittance. Therefore, the results in this work supplies a clue to synthesize oxygen-deficient VO_2_ related functional materials that have potentials applications in fabricating electronic devices and smart windows.

## Conclusion

In conclusion, W doped VO_2_ thin films with controlled oxygen vacancies were prepared by a modified sol-gel related mothed and followed by a post annealing under high vacuum. The films show much enhanced regulation performance in infrared transmittance as compared with conventional W-doping VO_2_ films. The enhanced infrared transmittance switching ability occurred at low temperature makes it as a suitable material for the applications to smart window coatings and novel electronic devices based on the thermochromic effects.

## Electronic supplementary material

Supplementary Information

## References

[1] Morin FJ (1959). Oxides Which Show A Metal-To-Insulator Transition At The Neel Temperature. Phys Rev Lett.

[2] Anderson, G. Studies on VO_2_. *Acta Chemic Scandiavica* **10** (1956).

[3] Marezio M, McWhan DB, Remeika JP, Dernier PD (1972). Structural Aspects of the Metal-Insulator Transitions in Cr-Doped VO_2_. Phys Rev B.

[4] Goodenough JB (1971). The two components of the crystallographic transition in VO_2_. J Solid State Chem.

[5] Bianconi A (1982). Multiplet Splitting Of Final-State Configurations In X-Ray-Absorption Spectrum Of Metal VO_2_: Effect Of Core-Hole-Screening, Electron Correlation, And Metal-Insulator-Transition. Phys Rev B.

[6] Christmann T, Felde B, Niessner W, Schalch D, Scharmann A (1996). Thermochromic VO_2_ thin films studied by photoelectron spectroscopy. Thin Solid Films.

[7] Shin S (1990). Vacuum-ultraviolet reflectance and photoemission study of the metal-insulator phase transitions in VO_2_, V_6_O_13_, and V_2_O_3_. Phys Rev B.

[8] Kang L, Gao Y, Luo H (2009). A novel solution process for the synthesis of VO_2_ thin films with excellent thermochromic properties. Acs Appl Mater Interfaces.

[9] Richardson MA, Coath JA (1998). Infrared optical modulators for missile testing. Optics & Laser Technology.

[10] Chen B, Yang D, Charpentier PA, Zeman M (2009). Al^3+^-doped vanadium dioxide thin films deposited by PLD. Sol Energ Mater Sol C.

[11] Marini, C. *et al*. Optical properties of V_1-x_Cr_x_O_2_ compounds under high pressure. *Phys Rev B* **77** (2008).

[12] Piccirillo C, Binions R, Parkin IP (2008). Synthesis and characterisation of W-doped VO_2_ by Aerosol Assisted Chemical Vapour Deposition. Thin Solid Films.

[13] Binions R, Piccirillo C, Parkin IP (2007). Tungsten doped vanadium dioxide thin films prepared by atmospheric pressure chemical vapour deposition from vanadyl acetylacetonate and tungsten hexachloride. Surface and Coatings Technology.

[14] Romanyuk A, Steiner R, Marot L, Oelhafen P (2007). Temperature-induced metal–semiconductor transition in W-doped VO_2_ films studied by photoelectron spectroscopy. Sol Energ Mater Sol C.

[15] Wang N, Liu S, Zeng XT, Magdassi S, Long Y (2015). Mg/W-codoped vanadium dioxide thin films with enhanced visible transmittance and low phase transition temperature. Journal of Materials Chemistry C.

[16] Tang C (1985). Local atomic and electronic arrangements in W_x_V_1-x_O_2_. Phys Rev B.

[17] Cao X, Wang N, Magdassi S, Mandler D, Long Y (2014). Europium Doped Vanadium Dioxide Material: Reduced Phase Transition Temperature, Enhanced Luminous Transmittance and Solar Modulation. Science of Advanced Materials.

[18] Aliev RA (2005). Effect of vacuum heat treatment on the metal-semiconductor phase transition in thin vanadium dioxide films. Tech Phys.

[19] Berezina O (2015). Vanadium oxide thin films and fibers obtained by acetylacetonate sol–gel method. Thin Solid Films.

[20] Zhang C, Cao W, Adedeji AV, Elsayed-Ali HE (2014). Preparation and properties of VO_2_ thin films by a novel sol–gel process. Journal of Sol-Gel Science and Technology.

[21] Wu J (2013). Effect of annealing temperature on thermochromic properties of vanadium dioxide thin films deposited by organic sol–gel method. Applied Surface Science.

[22] Zhang Y (2013). Direct preparation and formation mechanism of belt-like doped VO_2_(M) with rectangular cross sections by one-step hydrothermal route and their phase transition and optical switching properties. Journal of Alloys and Compounds.

[23] Binions R, Hyett G, Piccirillo C, Parkin IP (2007). Doped and un-doped vanadium dioxide thin films prepared by atmospheric pressure chemical vapour deposition from vanadyl acetylacetonate and tungsten hexachloride: the effects of thickness and crystallographic orientation on thermochromic properties. Journal of Materials Chemistry.

[24] Yan J, Huang W, Zhang Y, Liu X, Tu M (2008). Characterization of preferred orientated vanadium dioxide film on muscovite (001) substrate. Physica Status Solidi (A).

[25] Zhao Y (2012). Structural, electrical, and terahertz transmission properties of VO_2_ thin films grown on c-, r-, and m-plane sapphire substrates. J Appl Phys.

[26] Zhang J (2015). Self-Assembling VO_2_ Nanonet with High Switching Performance at Wafer-Scale. Chem Mater.

[27] Schilbe P (2002). Raman scattering in VO_2_. Physica B-Condensed Matter.

[28] Arcangeletti, E. *et al*. Evidence of a pressure-induced metallization process in monoclinic VO_2_. *Phys Rev Lett* **98** (2007). 10.1103/PhysRevLett.98.196406 17677642

[29] Radue E (2013). Effect of a substrate-induced microstructure on the optical properties of the insulator-metal transition temperature in VO_2_ thin films. J Appl Phys.

[30] Chen FH (2015). Control of the metal-insulator transition in VO_2_ epitaxial film by modifying carrier density. Acs Appl Mater Interfaces.

[31] Manning TD (2002). Intelligent window coatings: atmospheric pressure chemical vapour deposition of vanadium oxides. Journal of Materials Chemistry.

[32] Strelcov E (2016). Local coexistence of VO_2_ phases revealed by deep data analysis. Sci Rep.

[33] Silversmit G, Depla D, Poelman H, Marin GB, De Gryse R (2004). Determination of the V2p XPS binding energies for different vanadium oxidation states (V^5+^ to V^0+^). J Electron Spectrosc.

[34] Li W, Ji S, Qian K, Jin P (2015). Preparation and characterization of VO_2_(M)–SnO_2_ thermochromic films for application as energy-saving smart coatings. J Colloid Interface Sci.

[35] Ji S, Zhang F, Jin P (2011). Preparation of high performance pure single phase VO_2_ nanopowder by hydrothermally reducing the V_2_O_5_ gel. Sol Energ Mater Sol C.

[36] Warwick MEA, Ridley I, Binions R (2013). Thermochromic vanadium dioxide thin films from electric field assisted aerosol assisted chemical vapour deposition. Surface and Coatings Technology.

[37] Crane J, Warwick M, Smith R, Furlan N, Binions R (2011). The Application of Electric Fields to Aerosol Assisted Chemical Vapor Deposition Reactions. J Electrochem Soc.

[38] Zhang Y (2012). Preparation of W- and Mo-doped VO_2_(M) by ethanol reduction of peroxovanadium complexes and their phase transition and optical switching properties. Journal of Alloys and Compounds.

[39] Tan X (2012). Unraveling Metal-insulator Transition Mechanism of VO_2_ Triggered by Tungsten Doping. Sci Rep.

[40] Wu Y (2014). Depressed transition temperature of W_x_V_1-x_O_2_: mechanistic insights from the X-ray absorption fine structure (XAFS) spectroscopy. Physical Chemistry Chemical Physics: PCCP.

[41] Jeong J (2013). Suppression of metal-insulator transition in VO_2_ by electric field-induced oxygen vacancy formation. Science (New York, N.Y.).

[42] Appavoo K (2012). Role of defects in the phase transition of VO_2_ nanoparticles probed by plasmon resonance spectroscopy. Nano Lett.

[43] Li M (2014). Defect-mediated phase transition temperature of VO_2_ (M) nanoparticles with excellent thermochromic performance and low threshold voltage. Journal of Materials Chemistry A.

[44] Li Y, Liu Y, Liu J, Ren L (2016). The effects of niobium on the structure and properties of VO_2_ films. Journal of Materials Science: Materials in Electronics.

[45] Soltani M, Chaker M, Haddad E, Kruzelecky RV, Margot J (2004). Effects of Ti–W codoping on the optical and electrical switching of vanadium dioxide thin films grown by a reactive pulsed laser deposition. Applied Physics Letters.

[46] Chae BG, Kim HT (2010). Effects of W doping on the metal–insulator transition in vanadium dioxide film. Physica B: Condensed Matter.

[47] Griffiths CH, Eastwood HK (1974). Influence of stoichiometry on the metal‐semiconductor transition in vanadium dioxide. J Appl Phys.

[48] Lopez R, Haynes TE, Boatner LA, Feldman LC, Haglund RF (2002). Size effects in the structural phase transition of VO_2_ nanoparticles. Phys Rev B.

[49] Manning TD, Parkin IP, Pemble ME, Sheel D, Vernardou D (2004). Intelligent window coatings: Atmospheric pressure chemical vapor deposition of tungsten-doped vanadium dioxide. Chem Mater.

