# Supplementary material for: Synthesis and thermochromic property studies on W doped VO2 films fabricated by sol-gel method
Source: Sci Rep. 2017 Jul 21;7:6132. doi: 10.1038/s41598-017-05229-9 (PMC5522425; doi:10.1038/s41598-017-05229-9)
Supplement: Supplementary file 1 — Supplementary Information [file 41598_2017_5229_MOESM1_ESM.pdf]

Supplementary Information

for

Synthesis and thermochromic property studies on W doped VO_2 films fabricated by sol-gel method

Guoping Pan[1], Jinhua Yin[2], Keli Ji[2], Xiang Li[1, *], Xingwang Cheng[1], Haibo Jin[1] and Jiping Liu[1]

[1]Beijing Key Laboratory of Construction Tailorable Advanced Functional Materials and Green Applications, School of Materials Science and Engineering, Beijing Institute of Technology, Beijing, 100081, China.

[2]Physics Department, University of Science and Technology Beijing, Beijing, 100083, China.

[*]To whom should be corresponded: aihyoo@bit.edu.cn

The interaction between aqueous or alcohol Sol-Gel and substrate is weak. So the substrates were through special treatment before spin-coating[1-3] or the precursor could not be dispersed on the substrate uniformly to form fine pre-deposited film and the smooth VO_2 films were not obtained finally. The distilled water and ethyl alcohol were employed as the reaction solvent to prepared precursor as most previous works before using isobutanol solvent; however, the precursors were distributed on the substrate in small droplets after spin-coating and the final films show discontinuity, as shown in the Figure S1.

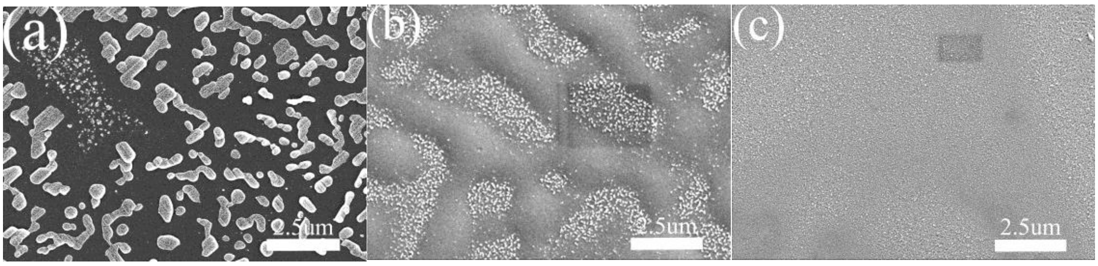

Figure-S1 The SEM images for 1% W-doped VO_2 films annealed at 470℃/4h from a) aqueous Sol-Gel, b) alcohol Sol-Gel and c) isobutanol Sol-Gel with the same

processing

From Figure-S1, there were some isolated large particles on the surface of VO_2 films by aqueous Sol-Gel and the surface become a little compact for VO_2 films by alcohol Sol-Gel. From S1 (c), the obtained VO_2 films by isobutanol Sol-Gel were dense and flat, which were superior to S1 (a) and (b) obviously.

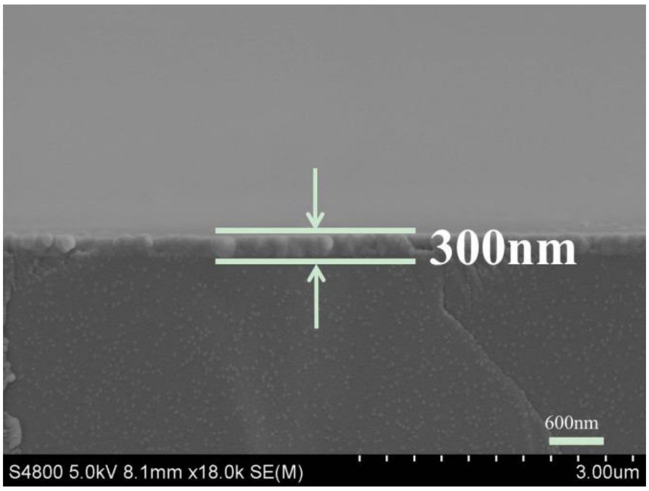

Figure-S2 The cross section SEM image for 1% W-doped VO_2 films annealed at 470℃ /4h

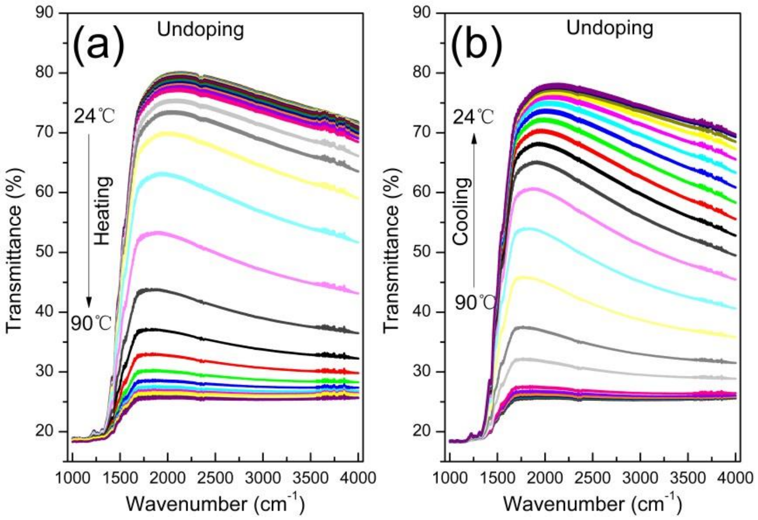

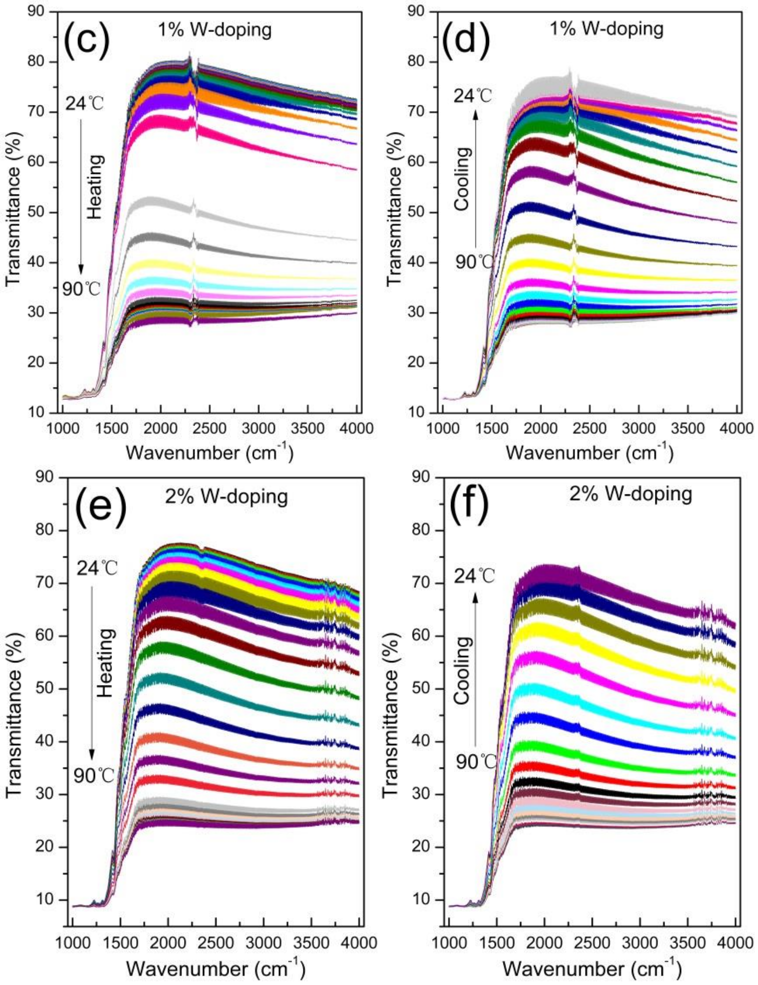

Figure-S3 The infrared transmittance curves of the undoped, 1%W and 2%W doped samples in the a), c), e) heating and b) d), f) cooling in the range of 1000-4000 cm^{-1}.

References

1 Monfort, O. *et al.* Reduction of V_2O_5 thin films deposited by aqueous sol–gel method to VO_2(B) and investigation of its photocatalytic activity. *Applied Surface Science* **322**, 21-27 (2014).

2 Lan, S.-D., Cheng, C.-C., Huang, C.-H. & Chen, J.-K. Synthesis of sub-10 nm VO_2 nanoparticles films with plasma-treated glass slides by aqueous sol–gel method. *Applied Surface Science* **357**, 2069-2076 (2015).

3 Chen, J.-K. *et al.* Using Solvent Immersion to Fabricate Variably Patterned Poly(methyl methacrylate) Brushes on Silicon Surfaces. *Macromolecules* **41**, 8729-8736 (2008).
